# Serosurvey of IgG Antibodies against *Bartonella henselae* and *Rickettsia typhi* in the Population of Attica, Greece

**DOI:** 10.3390/tropicalmed5030145

**Published:** 2020-09-16

**Authors:** Georgios Dougas, Maria Mavrouli, Athanassios Tsakris, Charalambos Billinis, Joseph Papaparaskevas

**Affiliations:** 1 Department of Microbiology, Medical School, National and Kapodistrian University of Athens, 11527 Athens, Greece; mmavrouli@med.uoa.gr (M.M.); atsakris@med.uoa.gr (A.T.); ipapapar@med.uoa.gr (J.P.); 2 Department of Microbiology and Parasitology, Faculty of Veterinary Science, University of Thessaly, 43100 Karditsa, Greece; cbillinis@gmail.com

**Keywords:** seroepidemiologic studies, serology, *Bartonella henselae*, *Rickettsia typhi*, fluorescent antibody technique, rats, Greece

## Abstract

*Rickettsia typhi and Bartonella henselae* are the causative agents of murine typhus and cat-scratch disease, respectively. A small-scale survey (N = 202) was conducted in the Attica region, Greece, for determining the prevalence rates of IgG antibodies against *B. henselae* and *R. typhi* by indirect fluorescence antibody test. IgG against *B. henselae* and *R. typhi* were present in 17.8% (36/202) and 4.5% (9/202) of the participants, respectively; co-occurring IgG against both *B. henselae* and *R. typhi* were detected in 3.5% (7/202), whereas only anti-*B. henselae* IgG in 14.3% (29/202), and only anti-*R. typhi* IgG in 1.0% (2/202). Titres 1/64, 1/128, 1/256, and 1/512, of anti-*B. henselae* IgG were identified in 6.4%, 4.5%, 4.5%, and 2.4%, whereas titres 1/40 and 1/80 of anti-*R. typhi* IgG were detected in 4.0%, and 0.5%, respectively. A positive association of anti-*B. henselae* IgG prevalence with a coastal area featuring a major seaport (*p* = 0.009) and with younger age (*p* = 0.046) was identified. The findings of this survey raise concern for exposure of the population of Attica to *B. henselae* and *R. typhi*, which should be considered in the differential diagnosis when compatible symptoms are present. Our results also suggest that seaports may represent high-risk areas for exposure to *Bartonella* spp.

## 1. Introduction

Among flea-borne pathogens, *Rickettsia typhi* and *Bartonella henselae* are causative agents of murine typhus and cat-scratch disease, well described disease entities, for which conventional serological tests are widely available [1].

Murine (endemic) typhus frequently appears as a non-eschar forming malaise, with variable manifestations commonly including fever, headache and rash [2]. Infection is frequently acquired through exposure to rat-fleas (*Xenopsylla cheopis*) [3,4]. However, an urban transmission cycle of *R. typhi*, involving companion animals, especially cats, and cosmopolitan pet flea species (*Ctenocephalides* spp.) has also been recognized [5].

Cat-scratch disease is manifested as a sub-acute febrile regional lymphadenopathy, usually spontaneously resolving from two weeks to four months. Immunocompromised patients may develop severe vasoproliferative tumor-like lesions. Chronic sequelae, such as endocarditis, arthritis, endophthalmitis, neuroretinitis, and neurologic disorders, may occur [6,7]. *B. henselae* is maintained in asymptomatic bacteremic cats for prolonged period and subsequently acquired by fleas during blood meals [6]. Transmission *of B. henselae* occurs through direct skin inoculation of the pathogen by cat claws; however, exposure to *Ctenocephalides* spp. has been strongly suggested as another potential route of infection [6,8,9]. *B. henselae* may cause chronic infections and the bacterial DNA has been detected in saliva of cats and dogs, nevertheless, other transmission pathways from those animal species to humans have been suggested but not demonstrated [1,8,10,11,12].

In this study, we examined sera of residents of Attica in Greece for IgG antibodies against *R. typhi* and *B. henselae* and investigated potential risk factors for association with seropositivity. 

## 2. Materials and Methods 

The Attica region comprises eight regional units with a total area of 3808.10 km^2^, encompasses Athens, the country’s capital and largest city, and has a population of 3,756,453. The participants of the study were recruited among Attica residents visiting primary care biopathology laboratories for routine check-up or referred by a physician, during a 23-months period (March 2017–January 2019). The examinees were informed for the purpose of the study and voluntarily consented in written form for inclusion, completed a questionnaire and provided a blood sample. 

The collected information included age, gender, location of residence, profession, farming or gardening activities, subjective perception of insect bites excluding mosquitoes, and contact with pets. Those having contact with pets were additionally surveyed for consistent implementation of flea control regime according to the attending veterinarian and visual detection of fleas on the animals. The purpose of visiting the biopathology laboratory and the health status of the participants, were not surveyed. 

Approximately two mL of separated sera from blood drawn from a venipuncture, after centrifugation at 4000 × *g* for at least five minutes at room temperature, was collected from each participant and was stored at −20 °C. Each sample was tested with two commercial indirect immunofluorescence antibody test (IFAT) for detecting IgG antibodies, against *B. henselae* and *R. typhi*, respectively (Vircell^™^, S.L., Granada, Spain). The tests were manufactured with *B. henselae* genotype I (ATCC 49882/ Houston-1 strain) and *R. typhi* (ATCC VR-738/ Philip. Strain 18), grown in Vero cells. Tests were performed according to the manufacturer’s instructions. The examined serum was subjected in two-fold dilutions and inspected under a UV microscope, at 400× magnification; an apple green fluorescence was indicative of a reactive serum dilution. The end point dilution demonstrating fluorescence was the outcome of the assay (titre). Dilutions started from as low as 1/40 and 1/64, reaching a theoretical maximum of 1/640 and 1/1024, for *R. typhi* and *B. henselae*, respectively. Positive and negative controls were included in each run. A second independent observer repeated the microscopy examination to minimize bias; potential conflicts were resolved by a third observer. Statistical analysis was performed with chi-square test, binomial logistic regression and t-test, as necessary, using the commercially available software IBM SPSS Statistics for Windows, Version 20.0 (Armonk, NY, USA). The alpha level was set to 5%. 

The study protocol was approved by the Bioethics Committee of the National and Kapodistrian University of Athens, Medical School (approval reference no: 1415022715/2015). 

## 3. Results

### 3.1. Study Subjects

Examined sera corresponded to 202 individuals with a male to female ratio of 0.64 (N = 202) and a mean age of 51 years (95%CI:49–54); six, 20, 67 and 109 of the participants were allocated to the age groups of 2–14, 15–29, 30–50 and >50 years, respectively (N = 201). 

The majority of the examinees resided in the regional unit of Central Athens (42.6%), followed by those residing in North Athens (27.7%), Piraeus (17.3%), South Athens (6.4%), East Attica (4.5%), West Attica (1.0%) and West Athens (0.5%) (N = 202); all residencies were located in urban areas of Attica, including the Athens metropolitan area. The regional unit of Islands (less than 2% of Attica population) was not represented in this study. 

Contact with companion animals was reported by 59.7% (120/201) of the participants; 24.9% (50/201) with dogs, 9.9% (20/201) with cats, 24.9% (50/201) with both dogs and cats; Among subjects with companion animal contact (N = 120), consistent administration of anti-flea treatment was implemented by 60.7% (71/117), whereas presence of fleas on the animals was perceived from 39.0% (46/118). Profession related with outdoor activities was reported by 5.1% (10/195). Gardening and/or farming was practiced by 39.0% (73/187). Insect bites, excluding mosquitoes, were subjectively perceived by 29.0% (58/200).

### 3.2. IgG Antibodies Prevalence Rates

IgG antibodies against *B. henselae* and *R. typhi* were identified in 17.8% (36/202) and 4.5% (9/202) of the participants, respectively. IgG solely against *B. henselae* were present in 14.3% (29/202), whereas solely against *R. typhi* in 1.0% (2/202). Co-occurring IgG against both *B. henselae* and *R. typhi* were detected in 3.5% (7/202). Titres 1/64, 1/128, 1/256, and 1/512, of anti-*B. henselae* IgG were identified in 6.4%, 4.5%, 4.5%, and 2.4%, whereas titres 1/40 and 1/80 of anti-*R. typhi* IgG were detected in 4.0%, and 0.5%, respectively (Table 1 and Supplementary Materials).

### 3.3. Risk Factors

**Figure 1:**
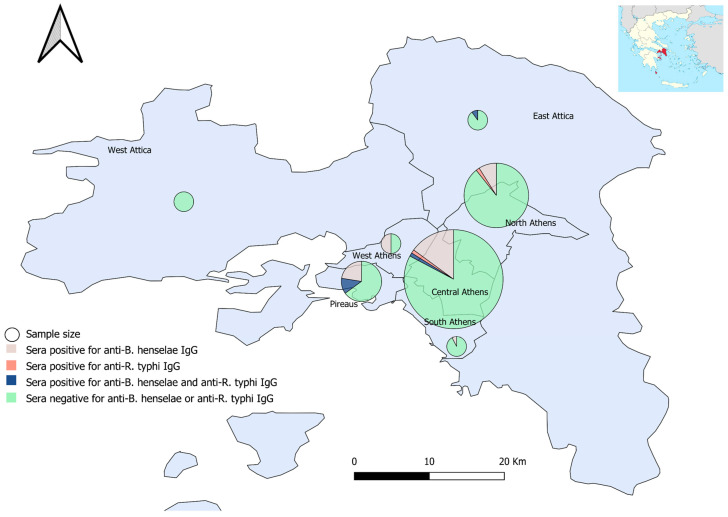
Sera sampling (size proportionate circles) and seropositivity percentages for anti-*B. henselae* and anti-*R. typhi* IgG antibodies, per Regional Unit, Attica, Greece.

The residents of the regional unit of Pireaus were associated with significantly increased seropositivity for *B. henselae* IgG (66.7%; 14/35) (χ^2^(6) = 17.737, *p =* 0.009). The highest prevalence for *R. typhi* IgG (14.3%; 5/35) was also observed in Pireaus; however, it did not reach statistical significance (*p* > 0.05) (Figure 1 and Supplementary Materials).

The *B. henselae* seropositive individuals had decreased mean age by 8.2 years (95%CI: −14.8 to −1.5) compared to seronegative ones (t (199) = 2.412, *p =* 0.017). A binomial logistic regression model revealed that for every additional year of age the likelihood for IgG antibodies detection was marginally decreased (OR = 0.976; 95%CI: 0.957 to 0.996), (χ^2^(1) = 5.705, *p* = 0.017). 

A tendency for cooccurrence of *R. typhi* and *B. henselae* was observed with higher odds for *R. typhi* seropositivity in individuals identified with *B. henselae* IgG than in seronegative ones (*p* = 0.011; OR = 19.8).

The prevalence of *R. typhi* IgG and the magnitude of titres for *B. henselae* or *R. typhi* were not affected by any of the examined factors. 

## 4. Discussion

In this serological survey we examined the prevalence and magnitude of IgG antibodies against *B. henselae* and *R. typhi*, using a commercial IFAT. This is the first study of IgG antibodies against *R. typhi* and *B. henselae* in Attica region in Greece, which includes the capital city of Athens.

The seroprevalence rates of IgG antibodies against *B. henselae* (17.8%) and *R. typhi* (4.5%) concur with previous studies conducted in the general population. Tea et al. [13] reported 19.8% seropositivity for *B. henselae* IgG in Greece. Variable rates were described in other countries; 15.0% in Korea, 23.0% in Poland, 9.6 to 19.6% in China, and 8.7% in Spain [14,15,16,17]. 

Anti-*R. typhi* IgG antibodies were identified at a rate of 2.0% in a previous study in Greece [18]. Rates of 3.9%, 1.3%, and 4.1% were reported in the Canary Islands of Spain, New Zealand, and China, respectively [19,20,21]. 

In our study, the prevalence of anti-*B. henselae* IgG antibodies was positively affected by younger age and a specific location of residence (Pireaus). A previous study in Greece reported increased seroprevalence at age groups of 2–14 and of 30–50 years [13] and thus concurs with our findings. 

In the present study, significantly increased IgG seroreactivity for *B. henselae* was detected in the regional unit of Pireaus, a coastal area of Attica, featuring the biggest passenger and commercial seaport in Greece. To our knowledge, no previous studies investigated residency at seaport areas as potential risk factor for human exposure to *Bartonella* spp. Kosoy et al. [22] suggested that influx of imported rats by ships causes a higher *Bartonella* spp. infection rate in rats located in seaports compared with the ones in mainland. *Xenopsylla cheopis*, the rat-flea, may harbor *Bartonella* spp. [23] and although with a preference for feeding from rats, it may readily bite humans. Furthermore, the most common flea, *Ctenocephalides felis* (cat flea), has been found to parasitize rats [24]. We hypothesize that frequent introduction of new infected rats in the seaport of Pireaus, leads to higher density of infectious fleas for *Bartonella* spp. and increased probability of human exposure. This is further supported by the fact that the animal-related factors of our study failed to explain antibody occurrence, suggesting a universal source of infection, such as the ubiquitous fleas. As IFAT is unable to distinguish *B. henseleae* from species commonly identified in rodents such as *B. tribocorum* or *B. elizabethae* [22,25], exposure cannot be attributed to specific *Bartonella* species. Rat population size, and other ecological factors may also have an impact in the flea-borne *Bartonella* spp. load. 

Our results did not support any modeling of anti-*R. typhi* IgG antibodies. However, residents of Pireaus presented the highest seroreactivity rate (*p* > 0.05), which is in agreement with previous reports of increased prevalence of seropositivity in human populations of seaport areas, mainly attributed to rat dynamics [26]. In a previous Greek study, higher mean age and agricultural professions were predisposing factors for anti-*R. typhi* IgG [18]. Bolaños-Rivero et al. reported an increased rate of *R. typhi* IgG to individuals older than 46 years and to those with farming activities [19]. However, our study subjects were mostly residents of urban areas with less chance of outdoor activities than the previously studied population. 

The participants of our study were recruited among people visiting biopathology laboratories. However, no information regarding health status or the purpose of visit was collected. As this study focused on IgG antibodies indicating past exposure, background health-related information or the reason of presentation to the laboratory would offer limited benefit and might introduce recall bias. Similarly, seeking volunteers among people presenting in laboratories for ordering diagnostic tests, would not introduce significant bias for the scope of the particular study. 

Regarding the used laboratory technique, IFAT, is considered the reference serology method for *B. henselae* and *R. typhi* [27], nevertheless, is subject to interpretation bias. We addressed this by validating the results by a second and in the case of disagreement, by a third examiner; however, no discrepancies occurred during the slide evaluation. However, IFAT is prone to false positives due to cross-reactions. Cross-reactivity of *B. henselae* with IgG antibodies against *Coxiella burnetii*, *Chlamydia pneumoniae, Erlichia chaffeensis* and non-henselae *Bartonella* [28,29], as well as between *R. typhi* and species of the typhus group or of the spotted fever group [30], has been well described. Discrimination among species is particularly important for *R. typhi* and *Rickettsia felis* or *Rickettsia felis-*like organisms, as the latter two are commonly present in *C. felis* fleas [30,31,32,33]. Western Blot assay and cross-absorption studies could assist in differentiating of IgG among *Bartonella* and *Rickettsia* species and could provide definite serological diagnosis [30,34]; however, due to limited resources, these techniques were not used in our study. 

Our observations are based on limited data and lack validation with additional confirmatory techniques; hence, more molecular and seroepidemiologic studies focused on *B. henselae* and *R. typhi*, and particularly the role of fleas in human exposure, are required. 

## 5. Conclusions

In summary, our results suggest exposure of the population of Attica to *B. henselae* and, to a lesser extent, to *R. typhi*. Seaports may represent high-risk areas for exposure to *Bartonella* spp. Physicians should consider *B. henselae* and *R. typhi* when evaluating patients with compatible symptoms. 

## Figures and Tables

**Table 1:** IgG antibodies against *Bartonella henselae* and *Rickettsia typhi* by Gender, Age Group and Contact with Companion Animals (N = 202).

*Anti-B. henselae* IgG							
	Titre						No (%)
	<1/64	1/64	1/128	1/256	1/512	1/1024	
Gender							
Male	64	4	6	4	1	0	15 (7.4)
Female	102	9	3	5	4	0	21 (10.4)
Age group							
≤14 years	4	0	2	0	0	0	2 (1.0)
15–29 years	16	2	0	2	0	0	4 (2.0)
30–50 years	50	6	4	4	3	0	17 (8.4)
>50 years	96	5	3	3	2	0	13 (6.4)
Contact with cats							
Yes	59	4	2	2	3	0	11 (5.4)
No	107	9	7	7	2	0	25 (12.4)
Contact with dogs							
Yes	87	4	3	3	3	0	13 (6.4)
No	79	9	6	6	2	0	23 (11.4)
Total No (%)	166 (82.2)	13 (6.4)	9 (4.5)	9 (4.5)	5 (2.4)	0 (0.0)	202 (100.0)
***Anti-R. typhi*** **IgG**							
	**Titre**						**No (%)**
	**<1/40**	**1/40**	**1/80**	**1/160**	**1/320**	**1/640**	
Gender							
Male	77	2	0	0	0	0	2 (1.0)
Female	116	6	1	0	0	0	7 (3.5)
Age group							
≤14 years	6	0	0	0	0	0	0 (0.0)
15–29 years	19	1	0	0	0	0	1 (0.5)
30–50 years	62	4	1	0	0	0	5 (2.5)
>50 years	106	3	0	0	0	0	3 (1.5)
Contact with cats							
Yes	67	3	0	0	0	0	3 (1.5)
No	126	5	1	0	0	0	6 (3.0)
Contact with dogs							
Yes	96	4	0	0	0	0	4 (2.0)
No	97	4	1	0	0	0	5 (2.5)
Total No (%)	193 (95.5)	8 (4.0)	1 (0.5)	0 (0.0)	0 (0.0)	0 (0.0)	202 (100.0)

## Supplementary Materials

The spreadsheet with the relevant with the study data of all participants is available online at https://www.mdpi.com/2414-6366/5/3/145/s1, Table S1: Supplementary data.
